# Penile Mondor’s disease after open hernia repair surgery: A case report

**DOI:** 10.5339/qmj.2024.38

**Published:** 2024-06-03

**Authors:** Riccardo Avantifiori, Giuseppe Cavallaro, Andrea Polistena, Luca Giordano, Giuliano D’Onghia

**Affiliations:** 1 Department of Surgery “Pietro Valdoni”, Sapienza University, Rome, Italy *Email: riccardo.avantifiori@gmail.com

**Keywords:** Penile Mondor’s Disease, hernia, Lichtenstein procedure, thrombophlebitis, sclerosis

## Abstract

**Background:**

Penile Mondor’s disease (PMD) is a rare syndrome characterized by sclerosis after superficial thrombophlebitis of the superficial penile veins. The most usual appearance of PMD is a tender, palpable, painful, and sometimes visible cord on the dorsal surface of the penis. Its pathogenesis is still unclear, and a standardized treatment has not been established.

**Case report:**

A 54-year-old male patient presented with a left-sided indirect reducible inguinal hernia. The patient underwent Lichtenstein’s procedure for inguinal hernia repair. On the tenth postoperative day, he returned with PMD confirmed by Doppler ultrasonography examination. Treatment with 4000 UI low molecular weight heparin (LMWH) daily for three weeks resolved the symptoms, but mild venous ectasia just to the proximal part of the penis remained.

**Discussion:**

The exact cause of PMD is not well understood, but various studies have identified certain factors associated with an increased risk of the condition. Out of various potential factors that could trigger PMD, the repair of an inguinal hernia has been reported only once. Treatment may involve pain management, anti-inflammatory medications, anticoagulants, and, in some cases, surgery.

**Conclusion:**

PMD after open hernia repair surgery is a very rare benign condition. Correct diagnosis and prompt treatment allowed symptom resolution. Residual venous ectasia has no clinical significance other than a cosmetic appearance.

## Introduction

Mondor’s disease (MD), first described in the early 1850s by Henri Mondor, is a rare entity of obliterative phlebitis involving the superficial veins on the thoracoabdominal wall.^1^ The exact mechanism of development of MD has not been established yet, but predisposing factors, including surgery, trauma, muscular strain, and breast cancer, have been presumed.^2^ The new immunohistochemical techniques showed that almost all MDs result from obstructed small blood vessels and, in some cases, occurred due to damaged lymph vessels.^3^

The most usual appearance of MD is a tender, palpable, painful, and sometimes visible cord on the thoracoabdominal wall. These symptoms usually resolve in approximately 2 to 8 weeks.

This rare clinical entity was also reported to have involvement of the superficial penile veins, called penile Mondor’s disease (PMD), first described by Hoffmann, counting to date about fifty cases described in the literature.^4,5^ The superficial penile vein is outside the deeper Buck’s fascia and is situated within the superficial dartos fascia, a continuation of the Colles fascia, on the dorsal surface of the penis. It opens into the superficial external pudendal vein, a tributary of the great saphenous vein. Among several factors potentially inducing PMD, inguinal hernia repair was, to the best of our knowledge, only once previously reported in a 32-year-old male with a right-sided inguinal hernia.^6^

We herein report a case of Penile Mondor disease that occurred after the Lichtenstein procedure was carried out for an inguinal hernia in our institution. We discuss this rare entity’s features, diagnostic, and treatment options. Written consent was obtained from the patient to publish this case report.

## Case Presentation

A 54-year-old male patient was referred by the family physician to Policlinico Umberto I – Rome, Italy, presenting with a left-sided indirect reducible inguinal hernia. The patient underwent a Lichtenstein tension-free mesh inguinal hernia repair procedure under local anesthesia (Mepivacaine 2%, a total of 20 ml) in a day-surgery setting. Lichtenstein procedure is considered the gold standard of hernia repair by the American College of Surgeons and is often used in our institution.^7^ No complication was reported during the procedure. He was discharged on the same day, and no adverse events were reported. On Postoperative Day (POD) 10, he returned to the outpatient clinic with cord-like induration on the dorsal surface of the penis and pain.

**Figure 1.**
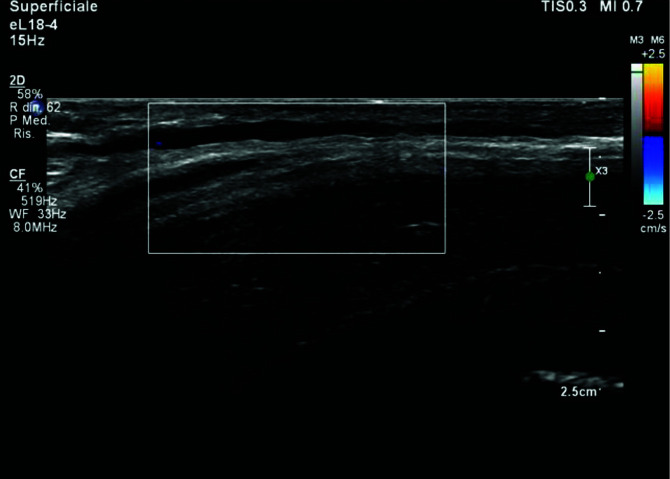
Superficial dorsal penile vein viewed with color doppler ultrasonography examination: no blood flow indicating thrombosis.

**Figure 2.**
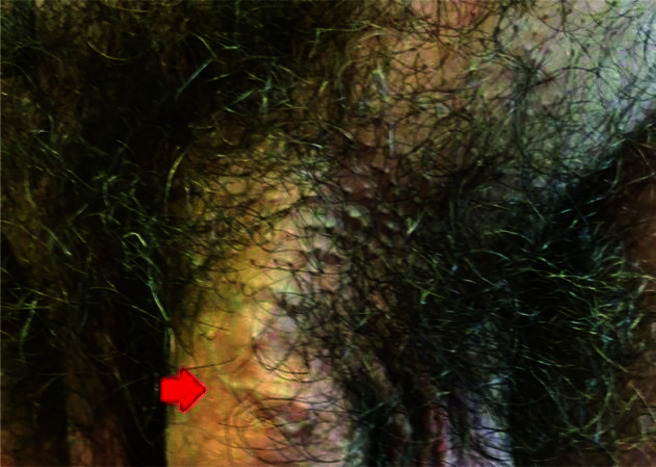
Dorsal surface of the penis after one year from diagnosis with residual mild venous ectasia just to the proximal part of the penis.

He was not taking any prescription medications; he was a non-smoker and had never lived with smokers. He had no known personal history of thromboembolic or hematologic disease, no history of penile trauma, no urogenital infection, and no history of prostate surgery. Full blood count, urea, electrolytes/creatinine, and fasting blood glucose were recorded in a normal range. No symptoms were declared except for pain during erections. We revealed subcutaneous cord-like induration of the superficial dorsal penile vein confirmed with a color Doppler ultrasonography examination (Figure 1). Diagnosis of PMD was made. He was treated with Paracetamol 1 g daily and 4000 UI low molecular weight heparin (LMWH) daily for three weeks. Improvement of symptoms during erections was noted. After one year, pain during erections completely disappeared, resulting in mild venous ectasia just to the proximal part of the penis (Figure 2).

## Discussion

MD can also involve the penis (PMD) and was initially reported by Hoffman in 1923, calling it “simulation of primary syphilis by gonorrheal lymphangitis”.^4^ Braun-Falco, in 1955, described penile thrombosis for the first time; later, Helm and Hodge reported the first case of superficial penile vein thrombosis.^8,9^ In 1996, it was termed Mondor’s disease of the penis.^10^

The pathogenesis of PMD remains unclear, but several studies have found some risk factors of PMD: vacuum erection device, penile trauma, strong sexual activity, prolonged erection, prolonged sitting position, hypercoagulation, urogenital infection, hematological disease, prostate biopsy and deficiency of S and C proteins and antithrombin III (found in 15%–20% of all families with thrombophilia).^11,12^

Due to the low incidence reported in the literature, a standardized management and treatment of PMD has not been established. Manimala et al., in a diagnostic and treatment algorithm for PMD, recommend excluding first an underlying malignancy and Peyronie’s Disease.^11^ Four to eight weeks of observation can be adopted, with the addition of non-steroidal anti-inflammatory drugs (NSAIDs) in case of pain. Then, a regular oral treatment with NSAIDs and/or LMWH can be administered. No antibiotic treatment is usually recommended. If a resolution with medical treatment is still not achieved, surgery through thrombectomy plus resection of the superficial penile vein may be considered.^13,14^ In a series of 25 patients with PMD, Al-Mwalad M. et al. reported that only two patients underwent surgery after non-resolution of symptoms after four weeks.^12^

Venous drainage of the penis is carried out by two systems: the superficial and deep dorsal venous systems. The anatomy of the superficial venous system of the penis is often variable. It allows blood to drain from the prepuce and skin of the penis and, running backward in the subcutaneous tissue, opens into the corresponding superficial external pudendal vein, a tributary of the great saphenous vein.^15^

In most instances, PMD typically resolves within four to six weeks.^13^ However, in our specific case, PMD following hernia repair using Lichtestein’s technique was alleviated with paracetamol and LMWH in three weeks, leaving behind some residual venous ectasia after one year.

## Conclusion

Superficial dorsal penile vein thrombosis, known as penile Mondor’s disease, is a rare benign and often completely resolving disease. Its pathogenesis is still unclear, and a standardized treatment has not been established. It is not fatal, but it may have a detrimental impact on the patient’s quality of life. To our knowledge, this is the second report on PMD after Lichtenstein’s hernia repair technique. Clinical examination with confirmation by ultrasonography is mandatory for diagnosis and correct management of PMD. In our case, a prompt non-operative treatment with paracetamol and LMWH allowed a symptom resolution with residual venous ectasia. Further studies are needed to better understand the underlying pathogenesis of this rare benign condition.

## Abbreviations

Mondor’s Disease (MD), Penile Mondor’s Disease (PMD), Postoperative Day (POD), low molecular weight heparin (LMWH), Non-Steroidal Anti-Inflammatory Drugs (NSAIDs).

## Conflict of Interest

The authors declare no conflict of interest.
